# Acute effects of euglycemic‐hyperinsulinemia on myocardial contractility in male mice

**DOI:** 10.14814/phy2.15388

**Published:** 2022-09-07

**Authors:** Satya Murthy Tadinada, Wojciech J. Grzesik, William Kutschke, Robert M. Weiss, E. Dale Abel

**Affiliations:** ^1^ Department of Neuroscience and Pharmacology, Carver College of Medicine University of Iowa Iowa City Iowa USA; ^2^ Fraternal Order of Eagles Diabetes Research Center, Carver College of Medicine University of Iowa Iowa City Iowa USA; ^3^ Abboud Cardiovascular Research Center, Carver College of Medicine University of Iowa Iowa City Iowa USA; ^4^ Division of Cardiology, Department of Internal Medicine, Carver College of Medicine University of Iowa Iowa City Iowa USA; ^5^ Division of Endocrinology and Metabolism, Department of Internal Medicine, Carver College of Medicine University of Iowa Iowa City Iowa USA; ^6^ Department of Medicine University of California Los Angeles Los Angeles California USA

## Abstract

Type 2 diabetes and obesity are associated with increased risk of cardiovascular disease, including heart failure. A hallmark of these dysmetabolic states is hyperinsulinemia and decreased cardiac reserve. However, the direct effects of hyperinsulinemia on myocardial function are incompletely understood. In this study, using invasive hemodynamics in mice, we studied the effects of short‐term euglycemic hyperinsulinemia on basal myocardial function and subsequent responses of the myocardium to β‐adrenergic stimulation. We found that cardiac function as measured by left ventricular (LV) invasive hemodynamics is not influenced by acute exposure to hyperinsulinemia, induced by an intravenous insulin injection with concurrent inotropic stimulation induced by β‐adrenergic stimulation secondary to isoproterenol administration. When animals were exposed to 120‐min of hyperinsulinemia by euglycemic‐hyperinsulinemic clamps, there was a significant decrease in LV developed pressure, perhaps secondary to the systemic vasodilatory effects of insulin. Despite the baseline reduction, the contractile response to β‐adrenergic stimulation remained intact in animals subject to euglycemic hyperinsulinemic clamps. β‐adrenergic activation of phospholamban phosphorylation was not impaired by hyperinsulinemia. These results suggest that short‐term hyperinsulinemia does not impair cardiac inotropic response to β‐adrenergic stimulation in vivo.

## Graphical abstract

Acute euglycemic hyperinsulinemia does not impair baseline cardiac function or its response to beta‐adrenergic stimulation induced inotropy.

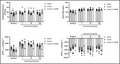

## 1 INTRODUCTION

Epidemiological data suggest that the risk of heart disease is higher in conditions with hyperinsulinemia such as Type 2 diabetes and obesity (Després et al., 1996; Perry et al., 1996). Despite these epidemiological correlates, the acute and chronic effects of hyperinsulinemia on cardiac function, are not well understood. In humans, euglycemic hyperinsulinemia increased heart rate, mean arterial pressure (Galderisi et al., 1991) and contractile performance (Rowe et al., 1981). However, these findings are challenged by other studies where no difference in inotropic state (Klein et al., 2010) or a chronotropic effect (Klein et al., 2010; Sasso et al., 2000) was observed during and after euglycemic hyperinsulinemia.

In animal models, early studies indicated that acute exposure to higher insulin levels resulted in increased force generation in ex vivo cardiac muscle strips from pigs (Airaksinen et al., 1985) and in dogs in vivo (Klinge & Wafin, 1971), although the inotropic state was apparent only upon blockade of β‐adrenergic receptors prior to application of insulin in a different study (Liang et al., 1982). Variable effects of insulin on inotropic responses of cardiac muscle in animal models have also been reported. Pharmacological doses of insulin were required to elicit chronotropy in isolated dog atria (Chiba, 1974), to increase cardiac contractility in dogs (Reikeras et al., 1985; Reikeras & Gunnes, 1986) and in neonatal pigs (Reiker et al., 1975). However, the magnitude of the myocardial effect of insulin in preparations with beta‐blockade was not consistent between studies (Chiba, 1974; Reiker et al., 1975).

In a recent study, insulin pretreatment was shown to attenuate the inotropic response to isoproterenol in Langendorff perfused mouse hearts (Fu et al., 2014). Mechanistically, this effect of insulin was mediated by G‐protein coupled Receptor Kinase 2 (GRK2) dependent phosphorylation of the β_2_‐adrenergic receptor, which primed the β_2_‐adrenergic receptor for coupling with Gα_i_ upon subsequent stimulation with β‐adrenergic receptor agonists. The switch in coupling of β_2_‐adrenergic receptor was sufficient to attenuate protein kinase A (PKA) activation following stimulation of β‐adrenergic receptors by isoproterenol (Fu et al., 2014). A similar attenuated response to catecholamines was also demonstrated previously in insulin pretreated myocardial preparations from dogs and pigs (Hiatt & Katz, 1969; Lee & Downing, 1976; Nudel et al., 1977).

The contribution of autonomic regulation in these ex vivo preparations cannot be evaluated, underscoring the need to evaluate the effect of insulin on inotropic responses in vivo. Whether insulin inhibits cardiac contractility induced by β‐adrenergic stimulation in an intact animal under euglycemic state has not been thoroughly assessed. Therefore, the goal of this study was to evaluate the in vivo effects of supraphysiological levels of insulin on contractility at baseline and following stimulation of cardiac β‐adrenergic receptors.

## 2 METHODS

### 2.1 Animals

All procedures involving the use of animals in this study were conducted in accordance with NIH guidelines for animal use in research and were approved by the Institutional Animal Care and Use Committee (IACUC) at the University of Iowa. Male C57BL6/J mice (stock: 000664) aged 6–8 weeks were purchased from Jackson Laboratories and were housed in the University of Iowa animal facility. Mice were maintained on a 12 h light:12 h dark cycle and had access to water and food ad libitum. For all experiments in this study, animals were fasted for 4–6 h on the day of experiment prior to starting all procedures (ZT0).

### 2.2 Reagents

Insulin (Humulin R‐100) was purchased from Eli Lilly, glucose (50%) was purchased from Hospira (Catalog #NDC0409‐6648‐02). Isoproterenol (Catalog# I6504) was purchased from Sigma. *N*ω‐nitro‐l‐arginine methyl ester (l‐NAME) hydrochloride was purchased from Cayman Chemicals (Catalog# 80210). pPLN S16 antibody (Catalog# A010‐12) was purchased from Badrilla, total PLN antibody (Catalog# ab2865) was purchased from Abcam. pAKT S473 antibody (Catalog# 4060S) and Pan AKT (Catalog# 2920S) were purchased from Cell Signaling Technology.

### 2.3 Intravenous injections

Insulin was injected at a dose of 1 U/kg into the right jugular vein of mice under isoflurane anesthesia (induction dose—5% v/v, maintenance dose—1.5% v/v). The injection volume was fixed at 20 μl and the control group received an equivalent volume of saline.

### 2.4 Hyperinsulinemic euglycemic clamps

The procedure for euglycemic hyperinsulinemic clamps has been previously described (Ayala et al., 2011). Briefly, mice were surgically implanted with a PE‐50 tubing catheter in the right external jugular vein and allowed to recover for 7–10 days. On the day of experiment, mice were fasted for 5–6 h and anesthetized using 5% (v/v) isoflurane anesthesia to test the patency of catheters and animals with patent catheters were used for experiments. Blood glucose measurements were made every 10 min beginning at 20 min prior to the start of infusions until 120 min after the infusion. At time *T*_0_, mice were infused with a bolus dose of 160 mU/kg insulin followed by 16 mU/kg/min insulin for 2 h concomitantly with a variable infusion rate of 50% dextrose solution to maintain euglycemia. In animals that received l‐NAME during the euglycemic‐hyperinsulinemic clamps, a bolus dose of l‐NAME (50 mg/kg) was injected intraperitoneally at T_0_, followed by a constant 1 mg/kg/min infusion until the end of the clamps.

### 2.5 Invasive hemodynamics

Cardiac function was assessed by cardiac catheterization in mice anesthetized using isoflurane anesthesia. A 1.4Fr catheter equipped with a pressure transducer (SPR‐671; Millar Instruments) was guided retrogradely into the left ventricle and pressure traces were recorded using Powerlab 8/30 series (AD Instruments). After acquiring baseline left ventricular (LV) pressures for 2 min, isoproterenol was injected intraperitoneally to assess changes in cardiac contractility. LabChart 8 software (AD Instruments) was used for data acquisition and analysis. Indices of contractile function including LV developed pressure, *dP*/*dt*_max_ and relaxation parameters including *dP*/*dt*_min_ were determined using the blood pressure plugin available within the LabChart software.

### 2.6 Western blots

Tris‐tricine gels (15%) were used for Phospholamban (PLN) immunoblots and 4%–12% Bis‐Tris gels (Invitrogen) were used for AKT immunoblots. Proteins were transferred to polyvinylidene difluoride membranes (Immobilon‐P; Sigma‐Aldrich) overnight at constant voltage (25 V) at 4°C. Primary antibodies were used at 1:2000 dilution (PLN^S16^) and 1:1000 dilution (pAKT^S473^, Pan‐AKT, and PLN) and blots were incubated overnight. Fluorophore‐conjugated secondary antibodies (Cell Signaling) at 1:10,000 dilution was used for detection using a LiCOR Odyssey scanner (LI‐COR). Images were quantified using Imago Studio Lite (LI‐COR) for densitometric analysis. Ratio of phosphorylated to total protein was used to adjust for variability in sample loading and quantified data were normalized to saline controls.

### 2.7 Statistical analysis

Data were analyzed using Graphpad Prism v8 software and presented as mean ± standard error of the mean. Hyperinsulinemic‐euglycemic clamp data were analyzed by unpaired *t*‐test. Cardiac function data was analyzed using repeated measures two‐way analysis of variance followed by post‐hoc analysis using Tukey's test for comparisons within the group and Sidak's test for comparisons between the groups. Significance is reported at *p* < 0.05.

## 3 RESULTS

### 3.1 Effect of insulin bolus on baseline cardiac function and responses to isoproterenol

**FIGURE 1:**
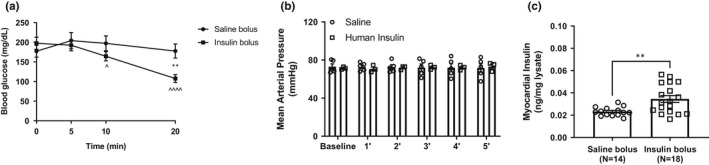
(a) Blood glucose in anesthetized mice injected with intravenous (i.v.) bolus of insulin (1 U/kg). (b) Mean arterial pressure measured in the carotid artery of animals injected with i.v. bolus of saline or insulin. (c) Myocardial insulin levels in animals subject to an i.v. bolus of saline or insulin (1 IU/kg). Data are presented as mean ± SEM. In (a) and (b), data were analyzed by repeated measures two‐way ANOVA followed by post‐hoc analysis using Tukey's test (within group comparisons) or Sidak's test (between group comparisons). Statistical significance was set at *p* < 0.05 (*N* > 4 animals/group; within group comparisons—**p < 0.01; ^*p* < 0.05, ^^^^*p* < 0.01 vs. saline). In (c), data were analyzed by student *t*‐test (**p < 0.01). ANOVA, analysis of variance; SEM, standard error of the mean.

**FIGURE 2:**
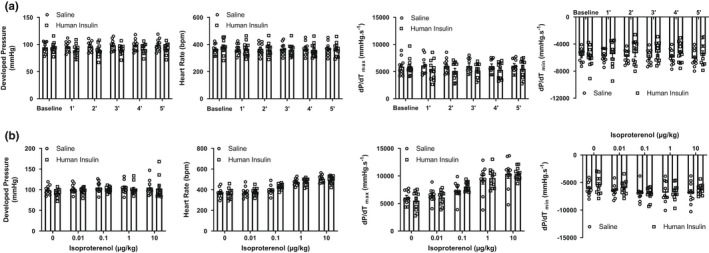
(a) Time dependent measures of cardiac function as determined by LV invasive hemodynamics in animals subject to intravenous (i.v.) bolus of saline or insulin. Data are presented as mean ± SEM and were analyzed by repeated measures two‐way ANOVA to determine the effect of insulin on cardiac function. Statistical significance was set at *p* < 0.05 (*N* > 10 animals/group). (b) Measures of cardiac function as determined by LV invasive hemodynamics in animals subject to i.v. bolus of saline or insulin followed by increasing doses of isoproterenol. Data are presented as mean ± SEM and were analyzed by repeated measures two‐way ANOVA to determine difference in inotropic response between groups. Statistical significance was set at *p* < 0.05 (*N* > 10 animals/group) and no significant differences in response to isoproterenol were present between the groups by Sidak's post‐hoc test. ANOVA, analysis of variance; LV, left ventricular; SEM, standard error of the mean.

**FIGURE 3:**
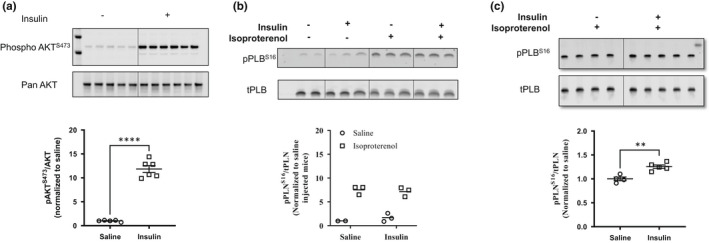
Myocardial phosphorylation of (a) AKT, (b) PLN in mice injected with intravenous (i.v.) bolus of saline or insulin (1 U/kg) and increasing doses of isoproterenol, (c) myocardial phosphorylation of PLN in animals injected with increasing doses of isoproterenol after euglycemic hyperinsulinemic clamps. Quantification of data in western blots is presented in graphs below. Data are presented as mean ± SEM. In (a) and (c) data were analyzed by student *t*‐ test (**p < 0.01, ****p < 0.0001). PLN, phospholamban; SEM, standard error of the mean.

To assess whether insulin directly influences baseline cardiac function of mice, we measured real‐time cardiac contractile function in stably anesthetized mice by LV invasive hemodynamics. We first established that there was no significant difference in blood glucose (Figure 1a) or in mean arterial pressure (as measured from the carotid artery, Figure 1b) between baseline and 5 min after intravenous (i.v.) insulin injection suggesting that insulin treatment did not cause hypoglycemia or alter systemic hemodynamics. Since insulin was delivered in this experiment as an i.v. bolus, we also measured insulin in the myocardium by enzyme linked immunosorbent assay and found an increase in myocardial insulin content (Figure 1c). This ensured the presence of insulin in the heart throughout the course of isoproterenol treatment. We assessed the effect of i.v. insulin injection on cardiac function (measured over 5 min) and found no significant changes in LV developed pressure or in contractile parameters (*dP*/*dt*_max_ and *dP*/*dt*_min_) (Figure 2a). To further assess the effect of insulin on β‐adrenergic receptor‐induced cardiac inotropy, we assessed myocardial contractility in response to increasing doses of isoproterenol delivered intraperitoneally starting at 5–7 min following i.v. injection of saline or insulin. Relative to baseline, intraperitoneal injections of isoproterenol in doses of 0.01 mg/kg to 10 mg/kg significantly increased the first derivatives of LV pressure and heart rates in both saline and insulin groups (Figure 2b). However, there was no significant difference between insulin and saline groups in terms of β‐adrenergic responsiveness at any dose of isoproterenol (0.01–10 mg/kg). These results suggest that acute exposure of the myocardium to a bolus injection of insulin does not modulate overall responsiveness to β‐adrenergic stimulation in vivo. In addition to elevated insulin levels in the myocardium (Figure 1c), insulin activation of myocardial insulin signaling was apparent, where phosphorylation of Akt on Ser^473^ was increased (Figure 3a). We also determined if acute exposure to insulin altered isoproterenol‐induced phosphorylation of the Ser^16^ residue of PLN, an effect that was previously described in vitro (Fu et al., 2014; Steinhorn et al., 2017). As shown in Figure 3b, phosphorylation of the Ser^16^ residue on PLN was not different in the hearts of saline versus insulin treated mice indicating a lack of difference in responsiveness to myocardial β‐adrenergic stimulation.

### 3.2 Effect of euglycemic‐hyperinsulinemia on baseline cardiac function and responses to isoproterenol

**FIGURE 4:**
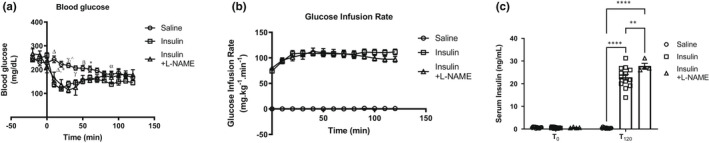
(a) Blood glucose levels, (b) glucose infusion rates and (c) serum insulin levels in animals subject to euglycemic‐hyperinsulinemic clamps. Data are presented as mean ± SEM. In (a), data were analyzed by two‐way repeated measures ANOVA followed by Tukey's post‐hoc test. Statistical significance was set at *p* < 0.05 (*N* > 4 animals/group; α, β, γ, Δ—saline vs. insulin, *, ^saline vs. insulin + l‐NAME; α, *p < 0.05, β, *p* < 0.01, γ, ^*p* < 0.001, Δ, *p* < 0.0001). In (c), data were analyzed by repeated measures two‐way ANOVA followed by post‐hoc analysis using Tukey's test (within group comparisons) or Sidak's test (between group comparisons). Statistical significance was set at *p* < 0.05 (*N* > 4 animals/group; between group comparisons—**p < 0.01, ****p < 0.0001). ANOVA, analysis of variance; l‐NAME, *N*ω‐nitro‐l‐arginine methyl ester; SEM, standard error of the mean.

**FIGURE 5:**
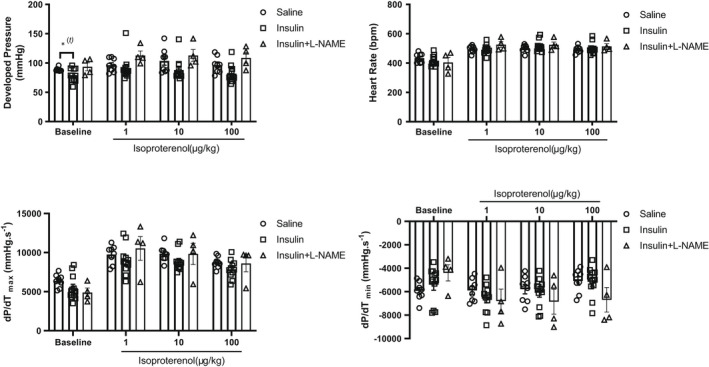
Measures of cardiac function as determined by LV invasive hemodynamics in animals subject to euglycemic‐hyperinsulinemic clamp followed by increasing doses of isoproterenol. Data are presented as mean ± SEM and were analyzed by repeated measures two‐way ANOVA to determine difference in inotropic response within and between groups. Statistical significance was set at *p* < 0.05 (*N* > 10 animals/group) and no significant differences in response to isoproterenol were present between the groups by Sidak's post‐hoc test. (*^(t)^ *p* < 0.05 by student's *t*‐test). ANOVA, analysis of variance; LV, left ventricular; SEM, standard error of the mean.

Since a bolus dose of i.v. insulin did not alter cardiac inotropy induced by β‐adrenergic stimulation and resulted in a decrease in blood glucose towards the end of the isoproterenol dose‐response (Figure 1a), we asked if a longer hyperinsulinemic state under euglycemic conditions alters myocardial β‐adrenergic responsiveness. We therefore, performed hyperinsulinemic‐euglycemic clamps, where euglycemic hyperinsulinemia was confirmed by stable blood glucose levels in mice subjected to a variable infusion of glucose and a constant infusion of insulin for 120 min. At the end of the clamp, proteins were isolated from hearts for PLN immunoblot and revealed a statistically increased phosphorylation of PLN at Ser^16^ in hearts subjected to hyperinsulinemia and isoproterenol exposure, relative to hearts that received isoproterenol alone (Figure 3c). Steady‐state glucose concentrations at the time of heart harvest were achieved by the 120‐min time point (Figure 4a). Given the possibility that hemodynamic measurements could be confounded by insulin‐induced peripheral vasodilation, mediated by endothelial nitric oxide synthase (eNOS) activation and nitric oxide (NO) production, we co‐infused animals with l‐NAME (50 mg/kg bolus i.p. followed by 1 mg/kg/min) for the duration of the clamps to determine whether the effect of hyperinsulinemia on LV developed pressure could be prevented. l‐NAME competitively inhibits eNOS and limits NO production. Interestingly, l‐NAME co‐infusion slightly reduced glucose infusion rates (Figure 4b) towards the end of the clamp and serum insulin levels were significantly increased relative to mice not receiving l‐NAME (Figure 4c). These data suggest a possible reduction in transendothelial transfer of insulin due to blockade of vasodilation by l‐NAME. Invasive hemodynamics under these conditions revealed a significant difference in LV developed pressure between the saline and insulin groups at the end of euglycemic hyperinsulinemia (Figure 5, LV developed pressure—saline vs. insulin: 88.7 ± 0.1 vs. 79.4 ± 2.4, *p* < 0.05 by *t*‐test). The effect of hyperinsulinemia on LV developed pressure was prevented in the l‐NAME group, but the modest but statistically insignificant effect of hyperinsulinemia on first derivatives of LV pressure (*dP*/*dT*_max_ and *dP*/*dT*_min_) persisted. These results indicate that despite normalizing LV developed pressure by preventing systemic vasodilation, supraphysiological hyperinsulinemia might modestly impair myocardial contractile function as shown by reduction in first derivatives of developed pressure. When mice were injected with increasing doses of isoproterenol, there was a significant increase in heart rate and first derivatives of LV pressure indicating equivalent inotropic responses in both the saline and insulin groups. l‐NAME treatment during the clamp did not attenuate the isoproterenol‐induced increases in contractility, indicating that the modest reduction in baseline contractile performance in l‐NAME treated animals might not be secondary to reduced responsiveness of the myocardium to basal catecholamine tone (Figure 5). Thus, the inotropic response following stimulation of the cardiac β‐adrenergic receptors is unaltered during steady‐state hyperinsulinemia.

Taken together, these findings suggest that supraphysiological level of insulin does not attenuate isoproterenol‐induced contractility and β‐adrenergic signaling in a physiological setting in vivo.

## 4 DISCUSSION

Epidemiological studies suggest that hyperinsulinemic states such as type 2 diabetes and obesity are associated with an increased risk of cardiovascular disease (Bella et al., 1998; Galderisi et al., 1991; Kannel & McGee, 1979). Exogenous insulin use, to maintain glycemia has also been associated with an increased risk of heart failure (Kannel et al., 1974; Nichols et al., 2004). These studies suggest that hyperinsulinemia may be an important contributor to the development of cardiac dysfunction in dysmetabolic states such as type 2 diabetes and obesity. However, the direct short‐term effects of hyperinsulinemia on cardiac function are relatively less well understood. To address this gap in knowledge, we measured cardiac contractile function by LV invasive hemodynamics in response to a bolus dose of insulin. Relative to baseline, a transient but statistically insignificant decrease in contractile performance was evident after insulin bolus. Since there was an apparent decline in cardiac function following insulin bolus, we studied whether exposure to insulin under euglycemic conditions of a longer duration would impact cardiac function. We therefore assessed LV developed pressure in mice subject to euglycemic‐hyperinsulinemic clamps and found a statistically significant reduction in baseline contractile parameters compared to control (saline) group. Although the baseline LV developed pressures were significantly lower in the insulin group compared to the control group, it was prevented by co‐infusion with l‐NAME to counter systemic vasodilatory effects of insulin. However, the trend towards reduced myocardial contractility in the insulin group persisted even when l‐NAME was used. These results suggest a potential direct but trivial effect of insulin on baseline cardiac function. To our knowledge, no studies have previously investigated the effect of hyperinsulinemia under euglycemic states in mice and our results suggest that under isoflurane anesthesia, euglycemic hyperinsulinemia does not significantly impact cardiac function over the course of 5 to 120‐min. Our results contrast with some previous reports on cardiac effects of insulin in other species. In healthy individuals, euglycemic hyperinsulinemia increased heart rate (Siani et al., 1990) and contractile function (Klein et al., 2010) but these findings have been challenged by other studies (Airaksinen et al., 1985; Lager et al., 1986; Sasso et al., 2000). In organ bath studies using papillary muscles from cats (Lee & Downing, 1976) and dogs (Lucchesi et al., 1972), and moderator bands (muscle bundles that link papillary muscles to the septum) from pigs (Lee & Downing, 1976), insulin treatment increased contractility. In dogs, intracoronary administration of insulin increased contractile function (Lucchesi et al., 1972) but the underlying molecular mechanisms mediating these responses are not understood. Lack of chronotropy or inotropy following insulin administration in our studies and the apparent differences in such effects reported in previous studies may be due to species related differences, in the source of insulin used or differences in innervation of the preparations and the consequences of autonomic reflexes. In a study using pig as a model, glucose–insulin–potassium (GIK) infusion exerted a positive effect on the relationship between myocardial oxygen consumption and contractile performance. This increased cardiac efficiency is reportedly mediated by an enhanced myocardial glucose oxidation but may also involve a contribution from decreases in vascular resistance since mean arterial pressures and end‐systolic pressure were lower when GIK infusion was used relative to intralipid‐heparin infusion (Korvald et al., 2000). Our data revealing no inotropic response of insulin infusion which contrasts with these pig studies. Our experimental protocol precluded the determination of substrate utilization and MVO_2_, thus it remains an open question if differences in the metabolic responses of these hearts to insulin could account for these differences.

Hyperinsulinemic states such as diabetes and obesity are associated with decreased cardiac reserve (Baldi et al., 2016; Pinto et al., 2014). In this context, the effect of acute hyperinsulinemia in modulating myocardial β‐adrenergic responsiveness has been previously assessed. The responses to increasing doses of isoproterenol were used to measure myocardial β‐adrenergic responsiveness in the present study. We found that acute hyperinsulinemia for durations of 5 min and 2 h does not attenuate cardiac contractile response to β‐adrenergic stimulation. These findings are in contrast with prior studies and the reasons for these discrepancies may include the species used for the study, altered milieu following abrogation of cephalic circulation (Nudel et al., 1977), anesthetic agent used and differences that may relate to the nature of preparation itself (in vivo vs. ex vivo).

Recent studies using isolated cardiomyocytes from adult mice showed altered cAMP dynamics and PKA activity when insulin pretreated myocytes were exposed to isoproterenol relative to myocytes treated with isoproterenol alone (Fu et al., 2014). Attenuation in isoproterenol induced PKA activity in insulin pretreated myocytes also correlated with reduced phosphorylation of PKA substrates such as Ser^16^ residue of PLN, which is important for regulating SERCA activity (Fu et al., 2014; Steinhorn et al., 2017). When we examined signaling changes following in vivo administration of isoproterenol in insulin treated mice, we did not observe a significant difference in Ser^16^ phosphorylation on PLN suggesting that insulin does not influence the biochemical or functional consequences of cardiac β‐adrenergic receptor activation under in vivo physiological settings.

## 5 LIMITATIONS

- Anesthesia affects cardiac function and therefore, the modest effects of insulin on myocardial function and myocardial β‐adrenergic responsiveness may or may not be relevant in the conscious state. Moreover, the studies were performed in animals with intact myocardial innervation. Thus, there is a possibility that the autonomic nervous system may have negated the potential direct effects on myocardial contractility which have been observed in ex vivo preparations.
- Insulin exposure is likely to alter myocardial substrate utilization and the impact of isoproterenol alone or in conjunction with insulin on substrate utilization was not determined. Therefore, we cannot rule out the possibility that subtle changes in myocardial substrate utilization and cardiac energetics could contribute to the modest change in first derivative of LV pressures in animals subject to euglycemic hyperinsulinemia. Evaluation of this possibility would require the use of metabolic tracers.
- All the experiments were performed with male mice to initially determine an interaction between insulin signaling and β‐adrenergic signaling to minimize potential interactions of hyperinsulinemic‐euglycemic clamp with estrous stage. Had a difference been observed, studies in female animals would have been performed. We therefore cannot rule out a possible interaction between insulin signaling and β‐adrenergic signaling in female mice.
- The present study does not assess the response of hearts to long‐term hyperinsulinemia as would occur following high‐fat feeding. Moreover, it does not address if hearts from insulin resistant mouse models would display a differential response to acute isoproterenol or insulin challenge under the conditions presented in this report.

In summary, results from our studies suggest that in mice subject to euglycemic hyperinsulinemia for 5–120 min, insulin does not exert a significant effect on cardiac function. In contrast to in vitro and ex vivo preparations, acute hyperinsulinemia up to 2 h does not attenuate myocardial contractility induced by β‐adrenergic stimulation.

## AUTHOR CONTRIBUTIONS

**E. Dale Abel** and **Satya Murthy Tadinada:** Conceptualization. **E. Dale Abel**, **Robert M. Weiss**, **Satya Murthy Tadinada:** Methodology. **Satya Murthy Tadinada**, **Wojciech J. Grzesik**, **William Kutschke:** Investigation. **Satya Murthy Tadinada:** Writing – original draft. **Satya Murthy Tadinada, E. Dale Abel:** Writing – review and editing. **E. Dale Abel**, **Robert M. Weiss:** Funding acquisition. **Robert M. Weiss**, **E. Dale Abel:** Resources. **E. Dale Abel:** Supervision.

## CONFLICTS OF INTEREST

The authors have no interest or relationships, financial or otherwise that represent a conflict of interest with the work described in this manuscript.

## ETHICS STATEMENT

All procedures involving the use of animals in this study were conducted in accordance with NIH guidelines for animal use in research and were approved by the Institutional Animal Care and Use Committee (IACUC) at the University of Iowa.
